# Aneuploidy shortens replicative lifespan in *Saccharomyces cerevisiae*

**DOI:** 10.1111/acel.12443

**Published:** 2016-01-13

**Authors:** Anna B. Sunshine, Giang T. Ong, Daniel P. Nickerson, Daniel Carr, Christopher J. Murakami, Brian M. Wasko, Anna Shemorry, Alexey J. Merz, Matt Kaeberlein, Maitreya J. Dunham

**Affiliations:** ^1^ Department of Genome SciencesUniversity of WashingtonFoege Building, Room S403B, 3720 15th Ave NE, Box 355065SeattleWA98195‐5065USA; ^2^ Departments of Biochemistry and Physiology and BiophysicsUniversity of WashingtonRoom HSB J‐355, 1705 NE Pacific St, UW box 357350SeattleWA98195‐7350USA; ^3^ Department of PathologyUniversity of WashingtonRoom HSB D‐514, 1705 NE Pacific St, Box 357470SeattleWA98195‐7470USA

**Keywords:** aging, aneuploidy, *BUL1*, protein trafficking, replicative lifespan

## Abstract

Aneuploidy and aging are correlated; however, a causal link between these two phenomena has remained elusive. Here, we show that yeast disomic for a single native yeast chromosome generally have a decreased replicative lifespan. In addition, the extent of this lifespan deficit correlates with the size of the extra chromosome. We identified a mutation in *BUL1* that rescues both the lifespan deficit and a protein trafficking defect in yeast disomic for chromosome 5. Bul1 is an E4 ubiquitin ligase adaptor involved in a protein quality control pathway that targets membrane proteins for endocytosis and destruction in the lysosomal vacuole, thereby maintaining protein homeostasis. Concurrent suppression of the aging and trafficking phenotypes suggests that disrupted membrane protein homeostasis in aneuploid yeast may contribute to their accelerated aging. The data reported here demonstrate that aneuploidy can impair protein homeostasis, shorten lifespan, and may contribute to age‐associated phenotypes.

## Introduction

Age is a major risk factor for some of the most deadly and debilitating human diseases, including heart disease, cancer, and Alzheimer's disease (Kaeberlein, 2013). Identifying and possibly intervening with the biological processes responsible for aging therefore has the potential to improve the healthspan of many individuals. Model organisms have been valuable to understanding the process of aging as several aging and longevity pathways are highly conserved (Smith *et al*., 2008). One such conserved pathway shown to play a role in aging is maintaining an appropriate balance between protein synthesis and protein degradation pathways in order to maintain a normal proteome, a process known as protein homeostasis (Powers & Balch, 2013; Labbadia & Morimoto, 2015). Maintaining protein homeostasis via upregulation of protein quality control systems, such as the ubiquitin‐proteasome system (UPS), has been shown to extend lifespan from yeast to rodents (Mehta *et al*., 2009; Kruegel *et al*., 2011; Rodriguez *et al*., 2012; Vilchez *et al*., 2012; Kaeberlein, 2013; Kevei & Hoppe, 2014). Inducing protein quality control systems is thought to extend lifespan by counteracting the age‐associated accumulation of damaged and aggregated proteins (Kevei & Hoppe, 2014); however, this age‐associated decline in proteostasis may be more finely regulated than currently appreciated (Labbadia & Morimoto, 2014). A second highly conserved process associated with longevity is caloric restriction and the corresponding decrease in signaling through the TOR complex (Johnson *et al*., 2013; Kaeberlein, 2013). Caloric restriction and inhibition of TOR by rapamycin have been shown to extend lifespan in yeast and metazoans (Smith *et al*., 2008).

Aneuploidy, defined as a number of chromosomes different from an integer multiple of the haploid complement, is another phenomenon that correlates with an organism's age: The prevalence of aneuploidy increases in both somatic and germline tissues as organisms age (Lushnikova *et al*., 2011; Nagaoka *et al*., 2012; Baker *et al*., 2013). While it is often proposed that the correlation between age and aneuploidy is due to increased rates of chromosomal missegregation in older cells, it is possible that the relationship is more complex and that aneuploidy itself contributes to aging phenotypes. Similar to old cells, aneuploid cells suffer from disrupted protein homeostasis due to uncompensated protein expression from the chromosome at altered copy number (Torres *et al*., 2007, 2010; Dephoure *et al*., 2014) and have increased levels of protein aggregates (Oromendia *et al*., 2012). Aneuploidy is further connected to aging by work in mice studying the effects of somatic aneuploidy on aging phenotypes. Mice have delayed aging phenotypes in tissues where the age‐associated rate of aneuploidy is also delayed (Baker *et al*., 2013). Conversely, mice with high levels of somatic aneuploidy due to mutations in the mitotic checkpoint protein BubR1 have decreased lifespans and premature aging phenotypes, while overexpression of BubR1 in mice decreases age‐associated somatic aneuploidy and extends lifespan (Baker *et al*., 2004, 2013). The human homolog of BubRI, *BUB1B*, is mutated in a rare human disorder known as mosaic variegated aneuploidy (MVA) syndrome (Matsuura *et al*., 2006); although individuals with this syndrome have limited life expectancies, they also have elevated rates of cancer and multiple congenital anomalies, making any assessment of an accelerated aging phenotype very difficult. Finally, accelerated aging has been observed in the most common example of human aneuploidy: Down syndrome (Martin, 1978; Roth *et al*., 1996; Nakamura & Tanaka, 1998). These diverse lines of evidence buttress the hypothesis that aneuploidy may not be only a symptom of aging but may also promote aging.

In this study, we examined the relationship between aneuploidy and aging in the yeast *Saccharomyces cerevisiae*. We found that aneuploid yeast generally have a shortened lifespan that correlates with the burden of extra yeast chromosomal DNA. We also showed that suppressor mutations could relieve this aneuploidy‐associated lifespan deficit. In particular, we showed that the loss of function of the gene *BUL1* rescued the lifespan of yeast with an extra copy of chromosome 5. The effect of *BUL1* deletion on lifespan was not generalizable to yeast disomic for other chromosomes or to euploid yeast. *BUL1* deletion also rescued a Cps1 trafficking defect in yeast disomic for chromosome 5; however, the precise mechanism by which *BUL1* deletion rescued the lifespan in chr5 disome strains remains uncertain. Therefore, while aneuploid cells are generally short lived, possibly secondary to perturbations in protein homeostasis, the precise perturbation, and therefore the precise mechanism of rescue, is likely karyotype dependent.

## Results

**Figure 1:**
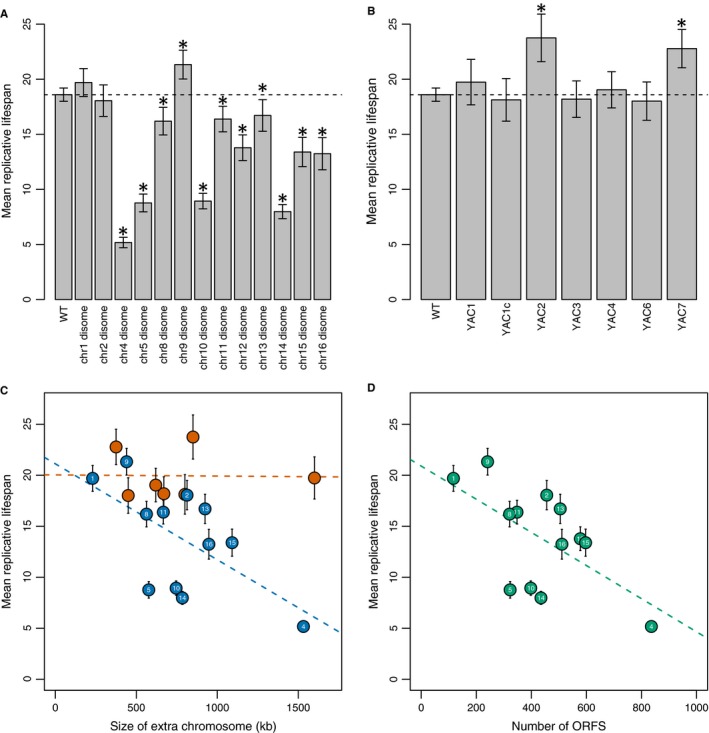
Aneuploidy decreases lifespan. (A) Aneuploidy of native yeast chromosomes decreases lifespan. Bars represent mean replicative lifespan ± 95% CI, *P < 0.001, Wilcoxon rank‐sum test. (B) The presence of native chromosome‐sized YACs does not decrease lifespan. Bars represent mean replicative lifespan ± 95% CI, *P < 0.001, Wilcoxon rank‐sum test. (C) The aneuploidy‐associated reduction in lifespan correlates with the size of the extra chromosome. Native yeast chromosomes = blue circles. Small numbers identify the chromosome number. *R* ^2^ = 0.38. YACs = red circles, *R* ^2^ = 0.00037. (D) The aneuploidy‐associated reduction in lifespan correlates with the number of ORFs duplicated. Native yeast chromosomes = green circles. Small numbers identify the chromosome number. *R* ^2^ = 0.36.

To investigate a causative relationship between aneuploidy and aging, we measured the replicative lifespans (RLS) of a set of previously well‐characterized disomic yeast strains (‘disomes’): haploid cells, each carrying an extra copy of one of 13 of 16 native yeast chromosomes (Table S1) (Torres *et al*., 2007, 2010; Sheltzer *et al*., 2011; Oromendia *et al*., 2012; Dephoure *et al*., 2014). We determined the RLS for each disome using the gold standard method of manually separating daughter cells as they bud from a single mother cell and counting the number of buds produced before a mother cell ceases to divide (Steffen *et al*., 2009). Using this method, we found that 10 of the 13 disomes assayed had significantly shorter lifespans than the wild‐type control strain (Figs 1A and S1).

Most transcripts and proteins originating from a disomic chromosome are expressed at levels reflecting their underlying DNA copy number (Torres *et al*., 2010; Dephoure *et al*., 2014). To differentiate whether the disomes’ shortened lifespan was due to this uncompensated overexpression, or simply to the burden of carrying an extra chromosome, we determined RLS for a collection of strains containing yeast artificial chromosomes (YACs) comprising human or mouse DNA. These YACs are similar in size to native yeast chromosomes but due to the different genetic density of mammalian genomes compared to *S. cerevisiae*, and the lack of appropriate splicing machinery in yeast, many fewer transcripts and proteins are likely expressed from YACs. Furthermore, previous comparisons between these disomes and YAC strains showed that the YAC strains did not suffer from the proteotoxic stress commonly experienced by disomes (Torres *et al*., 2007, 2010; Oromendia *et al*., 2012; Dephoure *et al*., 2014). Similarly, we found that while the disomes were short lived, strains carrying YACs even as large as 1.6 megabases had lifespans that were similar to wild‐type (Fig. 1B).

We noted that the degree of lifespan reduction in the disomes correlated with the size of the extra chromosome (*R* ^2^ = 0.38) (Fig. 1C). The size of the rDNA locus is highly variable between strains of yeast resulting in a wide variation in chromosome 12 size (James *et al*., 2009). Given this variability, we could not easily assign the exact size of chromosome 12 and chose to exclude chr12 disome from this analysis (for additional information, see Fig. S2). We also compared the RLS of each disome, including the chr12 disome, to the total number of verified ORFs present on each chromosome and found a similar correlation (*R* ^2^ = 0.36) (Fig. 1D); there was no correlation between the lifespan of the YAC strains and the size of the YAC (*R* ^2^ = 0.00037) (Fig. 1C).

**Figure 2:**
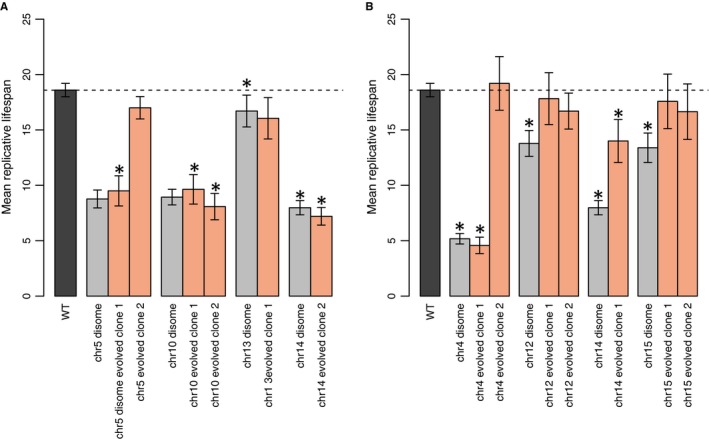
(A) Evolved disome clones with disomic karyotypes generally retained shortened lifespans. Chr5 disome evolved clone 2 was the only evolved disome clone with a disomic karyotype that had regained a wild‐type RLS. Bars represent mean replicative lifespan ± 95% CI,* *P *<* *0.001, Wilcoxon rank‐sum test. (B) Evolved disome clones that no longer contained a disomic karyotype generally had RLS closer to wild‐type. Bars represent mean replicative lifespan ± 95% CI,* *P *<* *0.001, Wilcoxon rank‐sum test.

All the disomes used in this study were previously shown to have a fitness deficit, as reflected in a lengthened doubling time and other phenotypes, compared to a euploid control (Torres *et al*., 2007). Previously, laboratory evolution of each disome selected for evolved clones with a rescued doubling time (Torres *et al*., 2010). To address whether the disomes’ shortened lifespans were simply due to a fitness deficit and not reflective of an aging phenotype *per se,* we determined the RLS of several fitness‐neutral evolved disome clones derived from disomes with shortened lifespans. As previously reported (Torres *et al*., 2010), these evolved disomes had diverse karyotypes containing both amplifications and deletions; these changes always reduced the overall DNA burden (Table S2). In general, we found that evolved disomes with the original disomic karyotype retained the short‐lifespan phenotype (Fig. 2A), while those clones that had a karyotype closer to the wild‐type euploid had regained normal lifespans (Fig. 2B). It is especially significant that most of the evolved disomes with a retained disomic karyotype (Fig. 2A) were still short lived despite regaining a normal doubling time as this strongly argues that the defective aging phenotype generally observed in the disomes cannot be explained simply by decreased growth ability.

**Figure 3:**
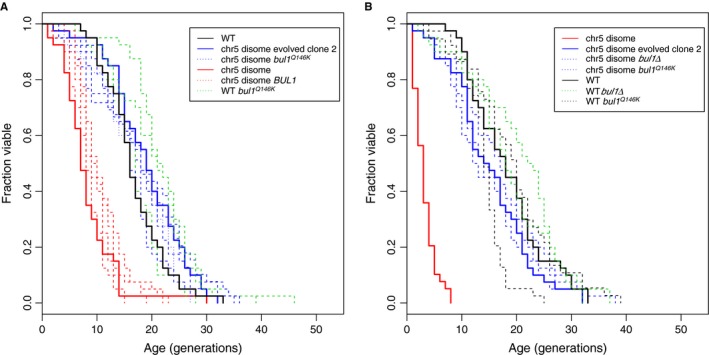
Mutations in *BUL1* rescue the lifespan defect in disomic yeast. (A) Segregants isolated from a backcross of chr5 disome evolved clone 2 have a normal lifespan if they carry the *bul1* ^Q146K^ mutation. Two of three euploid segregants carrying the *bul1* ^Q146K^ mutation show further lifespan extension beyond wild‐type. (B) A *bul1Δ* or *bul1* ^Q146K^ mutation is sufficient to rescue lifespan in the chr5 disomic background but does not further extend lifespan in a wild‐type background.

A single clone evolved from the chr5 disome (chr5 disome evolved clone 2) had both retained the disomic karyotype and had regained a wild‐type lifespan, suggesting that it had acquired a point mutation that suppressed the lifespan deficit associated with aneuploidy (Fig. 2A). Whole‐genome sequencing (WGS) of the original chr5 disome and the chr5 disome evolved clone 2 identified five point mutations unique to the evolved clone (Table S3). We backcrossed chr5 disome evolved clone 2 to an isogenic wild‐type strain and determined the replicative lifespan for nine segregants disomic for chromosome 5 and three euploid segregants. The RLS for the nine disomic segregants fell into two distinct groups: Four segregants had lifespans indistinguishable from the original chr5 disome, and five segregants had wild‐type lifespans (Fig. 3A). Sanger sequencing of the five evolved point mutations in these nine segregants revealed that a Q146K missense mutation in *BUL1* segregated exactly with the rescued lifespan (Fig. 3A, complete genotype details in Table S4). Two additional point mutations were identified on chromosome 5: a nonsense mutation in the dubious ORF *YER046W‐A* and an L59F missense mutation in *OXA1*. Although our experimental design did not allow us to segregate the two chromosome 5 point mutations away from the disomic chromosome, disomic segregants with both wild‐type and shortened lifespans were heterozygous for the chromosome 5 mutations, suggesting that these mutations were not crucial to affecting lifespan.

To test whether the *BUL1* variant alone was responsible for the lifespan extension, we introduced the *bul1* ^Q146K^ mutation into the original chr5 disome and observed a similar lifespan extension (Fig. 3B). As the Q146K mutation in Bul1 is just 11 residues upstream of the PPXY motif necessary for interaction with its E3 ubiquitin ligase partner, Rsp5 (Merhi & André, 2012), we created a *bul1Δ* in the chr5 disome and found that complete deletion of *BUL1* reproduces the lifespan extension (Fig. 3B). In a euploid background, *bul1* mutations had conflicting effects: The *bul1* ^Q146K^ variant extended lifespan beyond wild‐type in two of the three euploid segregants examined (Fig. 3A, Wilcoxon rank‐sum test *P* = 0.008 and 0.00006; Table S4 for genotype and RLS details). Generally, however, the lifespan‐extension effects of *bul1Δ* appear to be specific to the chr5 disome background. Neither the *bul1Δ* nor *bul1* ^Q146K^ allele significantly extended lifespan when reintroduced into the original wild‐type control (Fig. 3B), nor does *bul1Δ* extend lifespan in yeast disomic for other chromosomes (Fig. S3).

Previous characterization of the disome strains used in this study showed that these strains suffer from proteotoxic stress due to the uncompensated expression of proteins from the disomic chromosome (Torres *et al*., 2007, 2010; Oromendia *et al*., 2012; Dephoure *et al*., 2014). Specifically, the disome strains share an RNA expression profile similar to the environmental stress response and have an increased sensitivity to proteasome‐inhibiting drugs (Torres *et al*., 2007). Furthermore, deletion of the deubiquitinating enzyme Ubp6 increases proteasome degradation (Oromendia *et al*., 2012), rescues the fitness deficits of four disome strains (Torres *et al*., 2010), and ameliorates disome‐associated protein overexpression (Dephoure *et al*., 2014). Disome strains also have an increased level of protein aggregates (Oromendia *et al*., 2012). Together, these findings suggest that protein overexpression from the disomic chromosome increases flux through the UPS and results in a cellular stress response. While the degradation of damaged, misfolded, or misexpressed proteins by the UPS is the main pathway by which homeostasis of cytosolic proteins is maintained, integral membrane proteins do not typically have access to the UPS. Instead, damaged membrane proteins are targeted for endocytosis and pass through the multivesicular body (MVB) and to the lysosome (in yeast, the vacuole), where they are eventually degraded (Piper & Luzio, 2007; Zhao *et al*., 2013). Bul1 plays an important role in this plasma membrane quality control system by facilitating the Rsp5*‐*dependent polyubiquitination of multiple protein targets which directs their sorting to the vacuole for degradation (Yashiroda *et al*., 1996; De Craene *et al*., 2001; Helliwell *et al*., 2001; Crespo *et al*., 2004; Merhi & André, 2012; O'Donnell, 2012; Zhao *et al*., 2013). Disruption of this membrane protein quality control pathway has been previously linked to accelerated aging (Fabrizio *et al*., 2010), while the Rsp5/Bul1 node, in particular, has been shown to ameliorate the effects of protein aggregation in a model of neurodegenerative diseases (Tardiff *et al*., 2013).

**Figure 4:**
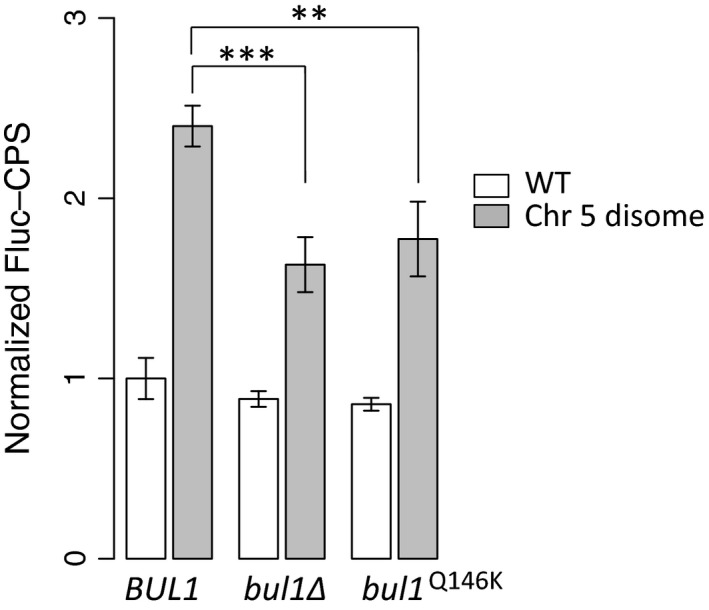
A mutation in *BUL1* rescues the trafficking defect in disomic yeast. Deletion of *BUL1* or the *bul1* ^Q146K^ allele rescues the disrupted MVB sorting present in yeast with a chr5 disome. A higher normalized Fluc‐CPS value indicates that cargo is not targeted efficiently to the MVB lumen. See Nickerson *et al*. (2012) for details (unpaired, two‐tailed *t*‐test, **P =* *0.002, ***P =* *0.0002). Bars represent means ± SD of 4 biological replicates each.

The vacuolar hydrolase, carboxypeptidase S (Cps1), is a transmembrane protein that requires Rsp5 for ubiquitination of its cytosolic domain and subsequent transport to the MVB lumen (Katzmann *et al*., 2004). Separate from its activity as an Rsp5 client, trafficking of Cps1 from the cell membrane to the vacuole has been well characterized and Cps1 is frequently used as a model cargo to study protein trafficking through MVBs (Odorizzi *et al*., 1998). Using a quantitative, luciferase‐based assay to search for defects in MVB formation and sorting (Nickerson *et al*., 2012; Nickerson & Merz, 2015), we observed that the original chr5 disome has a Cps1 trafficking defect that is ameliorated by the deletion of *BUL1* or the *bul1* ^Q146K^ allele (Fig. 4). We hypothesize that in cells disomic for chromosome 5, *BUL1* loss of function restores protein homeostasis by rescuing the membrane protein trafficking defect and allowing abnormally expressed proteins to be targeted to the vacuole for degradation.

## Discussion

Despite their temporal association, it has been unclear whether there is a causal relationship between aneuploidy and aging. Our results establish a connection between aneuploidy and aging. This connection cannot simply be explained by cellular fitness deficits associated with abnormal karyotypes as disomic yeast selected for normal growth rates generally retained shortened lifespans. The dose response between the burden of extra chromosomal DNA and lifespan deficit, although of only moderate strength, further supports the connection between aneuploidy and aging. Finally, the absence of abnormal aging phenotypes in yeast with a significant burden of exogenous DNA (i.e., YACs) implicates the expression of additional yeast proteins from native yeast chromosomes as a possible mechanism of the reduced lifespan in disomes. The data presented here add further support to the hypothesis that aneuploidy may not simply be a byproduct of an aging mitotic machinery but may also contribute to age‐associated phenotypes.

One possible explanation for the correlation between disome size and the aging deficit reported in this study is disrupted proteostasis. Strains disomic for larger chromosomes have been shown to overexpress a correspondingly greater number of proteins (Dephoure *et al*., 2014), which would therefore be expected to cause greater disruption to proteostasis. Disrupted protein homeostasis is thought to be a general process that contributes to aging in yeast and metazoans (Morimoto, 2008). In addition to their lifespan deficit, the disrupted protein trafficking phenotype observed in the chr5 disome strain is consistent with disrupted proteostasis. Furthermore, the rescue of both the aging deficit and the protein trafficking defect by a mutation in the ubiquitin ligase adaptor protein Bul1 implicates disrupted membrane proteostasis as a possible cause of the chromosome 5 disome's shortened lifespan. We have shown that the lifespan extension of *bul1Δ* is restricted to a chr5 disomic background. One possible explanation for this specific relationship is that Bul1's E3 ubiquitin ligase partner, Rsp5, is located on chromosome 5. It is possible that *bul1Δ* restores the balance of Rsp5‐mediated degradation by shifting its ubiquitination away from Bul1‐mediated proteins toward clients targeted by other arrestins, although this hypothesis remains to be tested explicitly. Interestingly, Rsp5 has previously been implicated in cellular tolerance of aneuploidy: A mutation in Rsp5 was identified in chr11 disome evolved clone 2 (Torres *et al*., 2010). As with all the evolved clones, this clone has a normal doubling time although the *RSP5* mutation was one of several mutations identified and its independent fitness effects remain unknown. However, *BUL1* deletion did not rescue the aging deficit found in the chr11 disome. Perturbations in the Rsp5/Bul1 protein degradation pathway have also been implicated in the tolerance to α‐synuclein, the protein that forms Lewy bodies in Parkinson's and other neurodegenerative diseases (Tardiff *et al*., 2013), suggesting perhaps that the Bul1 mutation could promote protein homeostasis via amelioration of the protein aggregate formation common to disome strains (Oromendia *et al*., 2012). A particular stoichiometry between Bul1 and Rsp5 may therefore be optimal for counteracting the disrupted protein homeostasis in aneuploid cells.

An alternative explanation for the correlation between disome size and the aging deficit is the copy number change of particular genes that affect aging in disomes. If we assume that genes with a dose‐dependent effect on aging are distributed randomly throughout the genome, then larger disomic chromosomes will be more likely to contain such genes and therefore more likely to cause severe aging deficits. In the specific case of chromosome 5 disomy, increased expression of particular chromosome 5 genes that disrupt nutrient signaling pathways could produce the observed aging deficit. Caloric restriction and decreased signaling through the TOR pathway is a conserved process that promotes longevity in yeast and metazoans (Kaeberlein, 2013). Gln3 is a transcription factor that regulates nitrogen metabolism, is directly regulated by both TOR and Bul1, and is located on chromosome 5. During growth on a nonpreferred nitrogen source, Bul1 and its functionally similar binding partner Bul2 are required for nuclear translocation of Gln3 (Crespo *et al*., 2004). Emphasizing the complexity of nutrient signaling, however, the inhibition of TOR by rapamycin treatment activates Gln3 via a mechanism that is not dependent on Bul1/Bul2 (Crespo *et al*., 2004). Bul1/Bul2 have additional roles in nutrient signaling upstream of Gln3 nuclear translocation. In response to poor nutrient conditions, such as growth on a nonpreferred nitrogen source, Bul1 and Bul2 are phosphorylated and inactivated. This allows the general amino acid transporter Gap1 to remain at the plasma membrane and scavenge for additional sources of nitrogen (Merhi & André, 2012; O'Donnell, 2012). Under nutrient‐replete conditions, signaling through TOR results in Bul1/Bul2 derepression, association with Rsp5, and resulting ubiquitination and endocytosis of Gap1 (Merhi & André, 2012; O'Donnell, 2012). Finally, additional support connecting Bul1/Bul2 function to aging via effects on nutrient signaling comes from a study where a natural variant of *BUL2* prolonged chronological aging in yeast via Gln3‐dependent effects on telomere length (Kwan *et al*., 2011). Clearly, Bul1/Bul2's role in mediating and responding to nutrient signaling is quite complex and its intricacies will likely require many future experiments to fully elucidate. However, two of Bul1/Bul2's partners in appropriate nutrient signaling are located on chromosome 5: *GLN3* and *RSP5*. Perhaps the particular stoichiometry of Bul1 to Gln3 or Rsp5 is restored by *bul1Δ* in the chr5 disome background resulting in lifespan extension. In contrast to the hypothesis outlined above linking the aging deficits in disomes to disrupted proteostasis, this hypothesis proposes that karyotype‐specific genes and pathways produce the aging deficits observed in each disome strain. In reality, the mechanism by which disomy affects cellular aging is likely a combination of karyotype‐specific effects as well as general changes in cellular physiology that represent shared characteristics of all disomes.

Although precise determination of the mechanism by which *bul1Δ* rescues lifespan in chr5 disome strains is beyond the scope of this paper, the results presented here provide a foundation for additional future experiments. First, although we have shown that *bul1Δ* in the background of chr5 disomy reproduces the lifespan rescue associated with the *bul1* ^Q146K^ mutation, confirmation of this genotype–phenotype connection could be even further strengthened by reintroducing *BUL1* in a chr5 disome *bul1Δ* background strain and assessing for recurrence of a shortened lifespan. Although theoretically simple, episomal plasmids are difficult to maintain in lifespan experiments and genomic integration into disome strains, which have increased mutation rates (Sheltzer *et al*., 2011), could result in additional suppressor mutations, making such an experimental approach fraught. Second, although previous studies have suggested that Bul1 and Bul2 are functionally redundant, we found that *bul1Δ* alone was sufficient to rescue lifespan in chr5 disomes. Future experiments will be important to determine the functional overlap between Bul1 and Bul2 and particularly in the area of longevity. Third, we have shown that the chr5 disome has a disrupted membrane trafficking phenotype that was rescued by *bul1Δ*. Future experiments will be needed to determine whether a similar membrane trafficking defect is a general phenotype of aneuploidy and if so, to discover both karyotype‐specific and general suppressors of this phenotype.

While this study shows that shortened lifespan is a common outcome of aneuploidy, it seems most likely that the specific mutations that suppress this phenotype depend on a cell's unique karyotype. Consistent with this hypothesis, the presence of *GLN3* and *RSP5* on chromosome 5 could explain the specific effects of *bul1Δ* on RLS in the chr5 disome background regardless of whether *bul1Δ* acts to rescue lifespan in chr5 disomes via restoration of protein homeostasis or through alterations in nutrient signaling. Cataloguing the finite list of mutations that act generally and karyotype specifically to suppress the cellular effects of aging will be an important step toward characterizing the mechanistic pathways by which aneuploidy perturbs cellular physiology. Such an effort will help define cellular targets and therapeutic approaches to age‐associated diseases including neurodegenerative diseases.

## Experimental procedures

### Media, strains, and primers

Disomes were grown on C‐his+G418 media to select for the disomic chromosome. Geneticin at a concentration of 200 mg L^−1^ was used to select for G418 resistance. YAC‐containing strains were grown on C‐ura‐trp media to select for the YAC. YPD, and sporulation media were prepared according to standard recipes as previously described. The original disome, evolved disome, and YAC‐containing strains used in this study were created and described previously (Torres *et al*., 2007, 2010) and are isogenic and of w303 background. The segregants derived from the backcross of chr5 disome evolved clone 2 were newly generated for this study. The genotypes for all strains and their strain identifiers are described in Table S1. The following primers were used to genotype the five point mutations identified in chr5 disome evolved clone 2 *BUL1*: F 5′‐TCGATGGGTGTGGACATTTT, R 5′‐ACCGTCGTTACACTCTCCAA; *BNR1*: F 5′‐, R 5′‐; *OXA1*: F 5′‐ CAAAACACTCTCAGCAGGCC, R 5′‐AGAGGGGAAACATCAGGCAT; *YER046W*: F 5′‐GAGTACTGAACGCACTGCAG, R 5′‐CTTTAGCGAAGGATGCTGCC; *YNL166C*: F 5′‐TGCGGTTTTGTGCTCTTCAA, R 5′‐ATACGGAGGATGTTGGCGAT.

*BUL1* deletion strains were created with standard methods as follows. For strains YMD2932, YMD2933, YMD2935, YMD2936, YMD2937, YMD2939, and YMD2942, the *bul1Δ::NatMX4* cassette was created by using primers BUL1:F and BUL1:R to amplify pRS40N plasmid DNA (Chee & Haase, 2012). This PCR product was then purified using Zymo columns and 2μg of the purified PCR product was used to chemically transform the following strains: w303 control, chr5 disome, chr8 disome, chr9 disome, and chr11 disome. Successful mutants were screened using primers PGO142 (5′‐ACTGGCGGTTTGTGGTTTGG) and PGO143 (5′‐GTGAAGTTGCATATAGCGGTAGCA) and karyotyped by aCGH. Microarray data are deposited in the Gene Expression Omnibus repository (http://www.ncbi.nlm.nih.gov/geo/) under accession GSE75623 and in the Princeton Microarray Database (http://puma.princeton.edu).

For strains YMD2934, YMD2938, YMD2940, YMD2941, YMD2943, YMD2944, YMD2945, the *bul1Δ::NatMX4* cassette was created by using primers BUL1:F and BUL1:R to amplify pRS40N plasmid DNA (Chee & Haase, 2012). This PCR product was then purified using Zymo columns and 2μg of the purified PCR product was used to chemically transform the w303 *MAT*a control (YMD671). Successful mutants were screened using primers PGO142 (5′‐ACTGGCGGTTTGTGGTTTGG) and PGO143 (5′‐GTGAAGTTGCATATAGCGGTAGCA) and backcrossed to w303 *MAT*α (YMD2566), sporulated, and selected for *MAT*α *bul1Δ::NatMX4* and histidine auxotrophy. This strain was then backcrossed to the appropriate disome strains and sporulated, and *MAT*a segregants were selected for growth on ClonNAT and C‐his plates. In the case of YMD2941, no *MAT*a segregants were identified so a *MAT*α strain was selected instead. Karyotypes were confirmed by aCGH. Microarray data are deposited in the Gene Expression Omnibus repository (http://www.ncbi.nlm.nih.gov/geo/) under accession GSE75623 and in the Princeton Microarray Database (http://puma.princeton.edu).

### Replicative lifespan experiments

Replicative lifespan assays were carried out as described previously (Steffen *et al*., 2009). All lifespan experiments were carried out on YPD with 2% agar. Disome and YAC‐containing strains requiring selective growth conditions were propagated on selective plates up until beginning the RLS. To start each lifespan, a small amount of cells was patched from the selective plates onto YPD and individual cells were arrayed. From these individual cells, virgin daughters were then picked to use for the RLS. Early control experiments showed that carrying out the RLS under nonselective conditions did not lead to a significant loss of the disomic chromosome (data not shown).

### Illumina sequencing analysis

DNA samples from chr5 disome and chr5 disome evolved clone 2 were sequenced on an Illumina MiSeq generating 4832685 or 2691304 150 bp reads leading to a mapping coverage of 94X and 52X, respectively. Sequencing data are deposited at BioProject ID PRJNA255436 with BioSample IDs SAMN02919171 and SAMN02919184. Reads were aligned with BWA (Li & Durbin, 2009), and single nucleotide variants (SNVs) were called using the samtools (Li *et al*., 2009) mpileup command after applying standard filters. Specifically, nonuniquely mapping reads, reads in which the pair did not map, reads with a mapping quality <30, and PCR/optical duplicate reads were filtered out; the samtools C‐50 filter was applied as recommended for reads mapped with BWA and the vcfutils.pl varFilter ‐D filter was applied with D set to 2X the average mapping coverage. SNVs unique to the evolved clones were identified with a custom Python script, annotated with a second Python script (http://depts.washington.edu/sfields/software/annotate/) (Pashkova *et al*., 2013) and further prioritized by manual examination with the Integrative Genome Viewer (IGV) (Robinson *et al*., 2011).

### LUCID cargo trafficking assay

Endosomal luminal cargo transport was measured using a chimeric luciferase reporter and a dual luciferase assay (Promega) essentially as described (Nickerson *et al*., 2012). Cells were grown to late log phase in selective media to ensure the retention of reporter plasmid pDN278 (Nickerson & Merz, 2015), which encodes a chimeric luminal cargo, *FLuc‐CPS1*, under the native *CPS1* promoter, and a soluble loading control, *Renilla*Luc, under the constitutive *PGK1* promoter in a pDN616 backbone (Nickerson *et al*., 2012). Measured sequentially in the same yeast cell lysate, signal from FLuc‐tagged cargo is normalized vs. signal from RLuc.

## Funding

This work was supported by NIH Grants R01 GM094306 to MJD, R01AG039390 to MK, and the University of Washington Nathan Shock Center of Excellence in the Basic Biology of Aging (NIH P30AG013280). BMW was supported by NIH Training Grant T32ES007032. AJM and DPN were funded by NIH grant GM077349. ABS was supported by F30 CA165440 and NIH Training Grant T32 AG000057 and is an ARCS Scholar alumnus. MJD is a Rita Allen Foundation Scholar and a Senior Fellow in the Genetic Networks program at the Canadian Institute for Advanced Research.

## Conflict of interest

None declared.

## Author contributions

ABS, DPN, AJM, MJD, and MK designed the experiments and analyzed the results. DC, DJM, BMW, and AS performed the replicative lifespan experiments. GTO constructed the *bul1Δ* strains. DPN carried out the trafficking experiments. ABS performed the statistical analyses and wrote the manuscript with input from MJD, MK, and AJM.

## Supporting information


**Fig. S1** Individual Kaplan–Meir curves for each strain compared to wild‐type control (w303). *P*‐values were determined by a Wilcoxon Rank‐Sum test.

**Fig. S2** The estimated size of chromosome 12 affects the correlation between disome size and mean RLS. The median, minimum, and maximum size of chromosome 12 as observed by (James *et al*., 2009) are plotted in S3A, S3B, and S3C respectively. A duplicate of the disome data presented in Fig. 1C is reproduced in S3D for comparison: in this case chr12 disome strain was removed from the comparison.

**Fig. S3** The lifespan extension seen in chr5 disomes with *bul1Δ* is not generalizable to other disomic backgrounds. Each bar represents the mean replicative lifespan ± 95% CI. All lifespans were performed on MATa clones except for chr 10 disome bul1Δ which is MATα; there is typically no difference in lifespan between MATa and MATα clones (Kaeberlein *et al*., 2005), Dark grey = WT control, light grey = disome, mint green = disome, bul1Δ.

**Table S1** Strains used in this study.
**Table S2** Change in DNA and gene content of evolved disomes relative to the original disomes.
**Table S3** Mutations discovered by WGS of chr5 disome evolved clone 2.
**Table S4** Complete genotypes of chromosome 5 disome evolved clone 2 segregants.
**Table S5** Replicative lifespan data summary.
**Table S6** Replicative lifespan raw data.
