# Supplementary material for: Aneuploidy shortens replicative lifespan in Saccharomyces cerevisiae
Source: Aging Cell. 2016 Jan 13;15(2):317–24. doi: 10.1111/acel.12443 (PMC4783355; doi:10.1111/acel.12443)
Supplement: Supplementary file 1 — Fig. S1 Individual Kaplan–Meir curves for each strain compared to wild‐type control (w303). P‐values were determined by a Wilcoxon Rank‐Sum test. [file ACEL-15-317-s001.pdf]

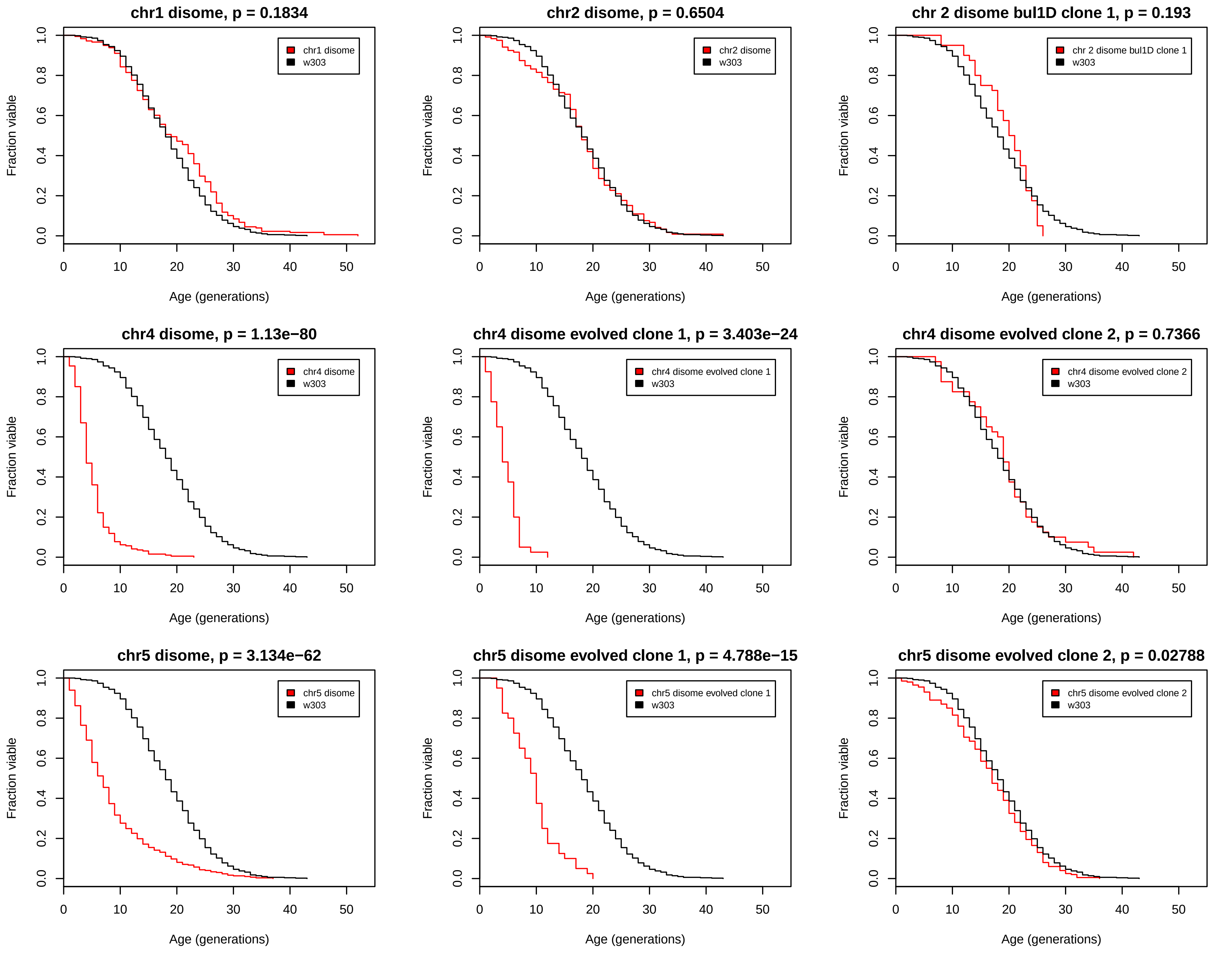
chr1 disome, p = 0.1834
chr1 disome
w303
chr2 disome, p = 0.6504
chr2 disome
w303
chr 2 disome bul1D clone 1, p = 0.193
chr 2 disome bul1D clone 1
w303
chr4 disome, p = 1.13e−80
chr4 disome
w303
chr4 disome evolved clone 1, p = 3.403e−24
chr4 disome evolved clone 1
w303
chr4 disome evolved clone 2, p = 0.7366
chr4 disome evolved clone 2
w303
chr5 disome, p = 3.134e−62
chr5 disome
w303
chr5 disome evolved clone 1, p = 4.788e−15
chr5 disome evolved clone 1
w303
chr5 disome evolved clone 2, p = 0.02788
chr5 disome evolved clone 2
w303
Fraction viable
Age (generations)
0.0
0.2
0.4
0.6
0.8
1.0
0
10
20
30
40
50

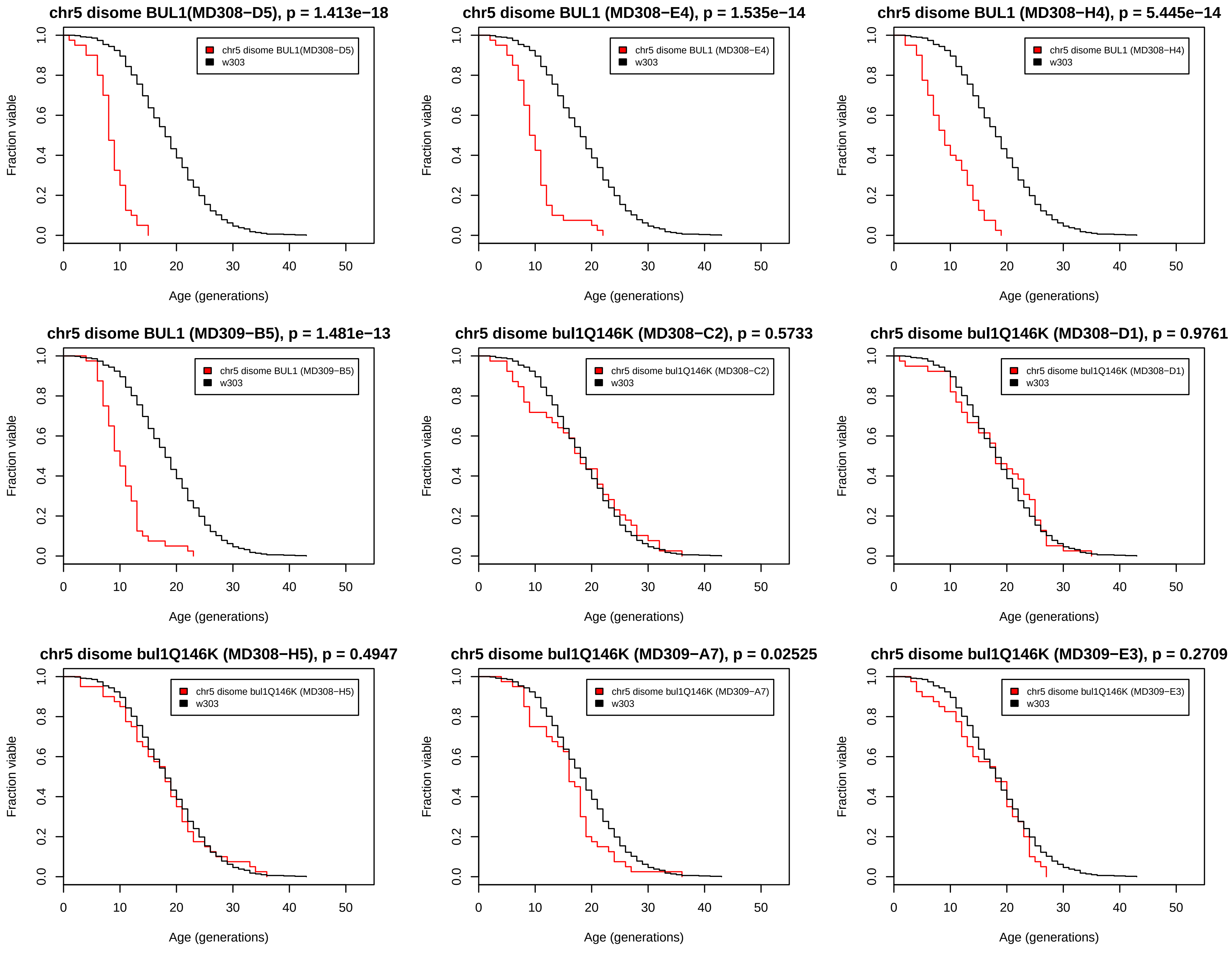
chr5 disome BUL1(MD308−D5), p = 1.413e−18
chr5 disome BUL1(MD308−D5)
w303
Fraction viable
Age (generations)
chr5 disome BUL1 (MD308−E4), p = 1.535e−14
chr5 disome BUL1 (MD308−E4)
w303
Fraction viable
Age (generations)
chr5 disome BUL1 (MD308−H4), p = 5.445e−14
chr5 disome BUL1 (MD308−H4)
w303
Fraction viable
Age (generations)
chr5 disome BUL1 (MD309−B5), p = 1.481e−13
chr5 disome BUL1 (MD309−B5)
w303
Fraction viable
Age (generations)
chr5 disome bul1Q146K (MD308−C2), p = 0.5733
chr5 disome bul1Q146K (MD308−C2)
w303
Fraction viable
Age (generations)
chr5 disome bul1Q146K (MD308−D1), p = 0.9761
chr5 disome bul1Q146K (MD308−D1)
w303
Fraction viable
Age (generations)
chr5 disome bul1Q146K (MD308−H5), p = 0.4947
chr5 disome bul1Q146K (MD308−H5)
w303
Fraction viable
Age (generations)
chr5 disome bul1Q146K (MD309−A7), p = 0.02525
chr5 disome bul1Q146K (MD309−A7)
w303
Fraction viable
Age (generations)
chr5 disome bul1Q146K (MD309−E3), p = 0.2709
chr5 disome bul1Q146K (MD309−E3)
w303
Fraction viable
Age (generations)

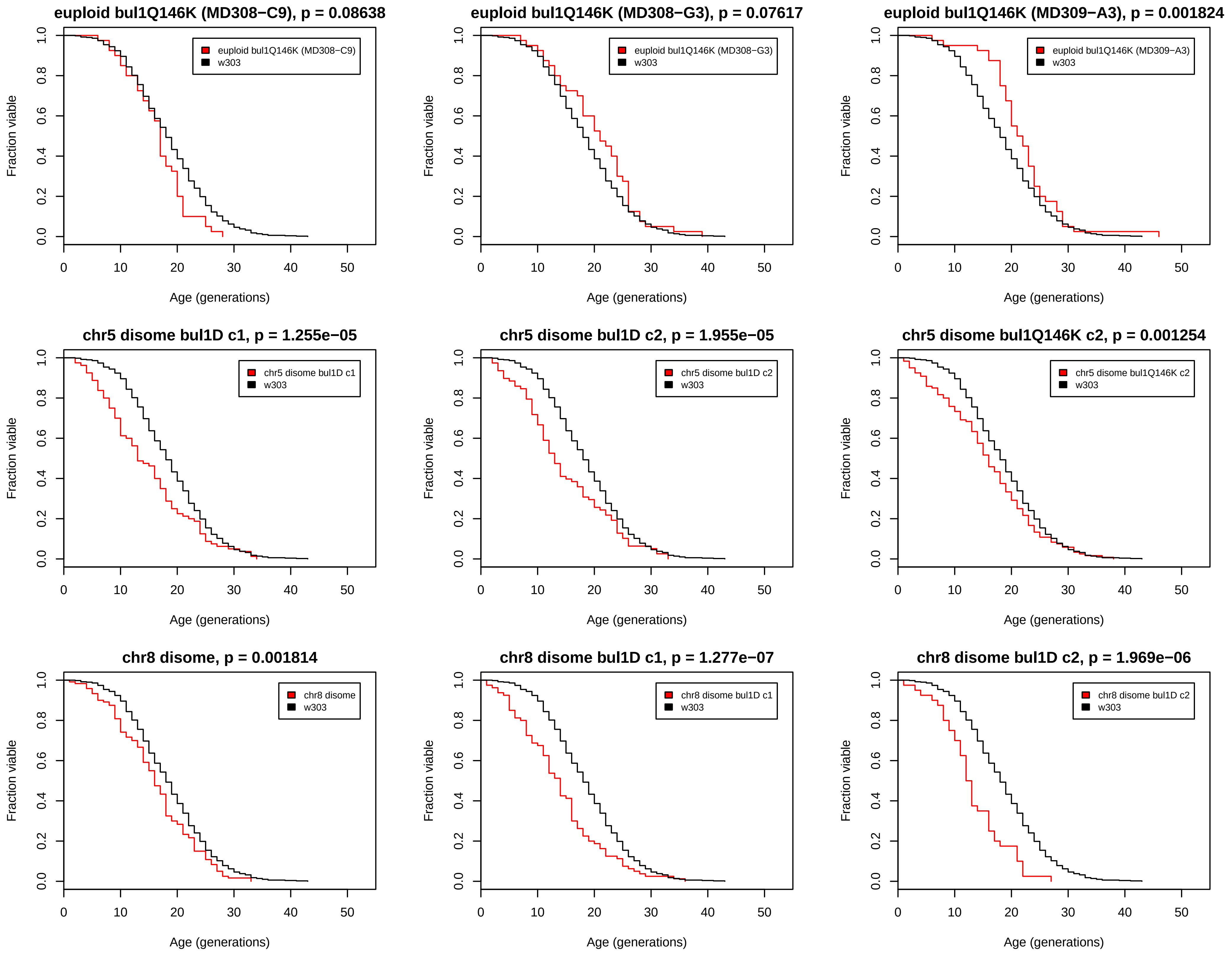
euploid bul1Q146K (MD308−C9), p = 0.08638
euploid bul1Q146K (MD308−C9)
w303
euploid bul1Q146K (MD308−G3), p = 0.07617
euploid bul1Q146K (MD308−G3)
w303
euploid bul1Q146K (MD309−A3), p = 0.001824
euploid bul1Q146K (MD309−A3)
w303
chr5 disome bul1D c1, p = 1.255e−05
chr5 disome bul1D c1
w303
chr5 disome bul1D c2, p = 1.955e−05
chr5 disome bul1D c2
w303
chr5 disome bul1Q146K c2, p = 0.001254
chr5 disome bul1Q146K c2
w303
chr8 disome, p = 0.001814
chr8 disome
w303
chr8 disome bul1D c1, p = 1.277e−07
chr8 disome bul1D c1
w303
chr8 disome bul1D c2, p = 1.969e−06
chr8 disome bul1D c2
w303
Fraction viable
Age (generations)
0.0
0.2
0.4
0.6
0.8
1.0
0
10
20
30
40
50

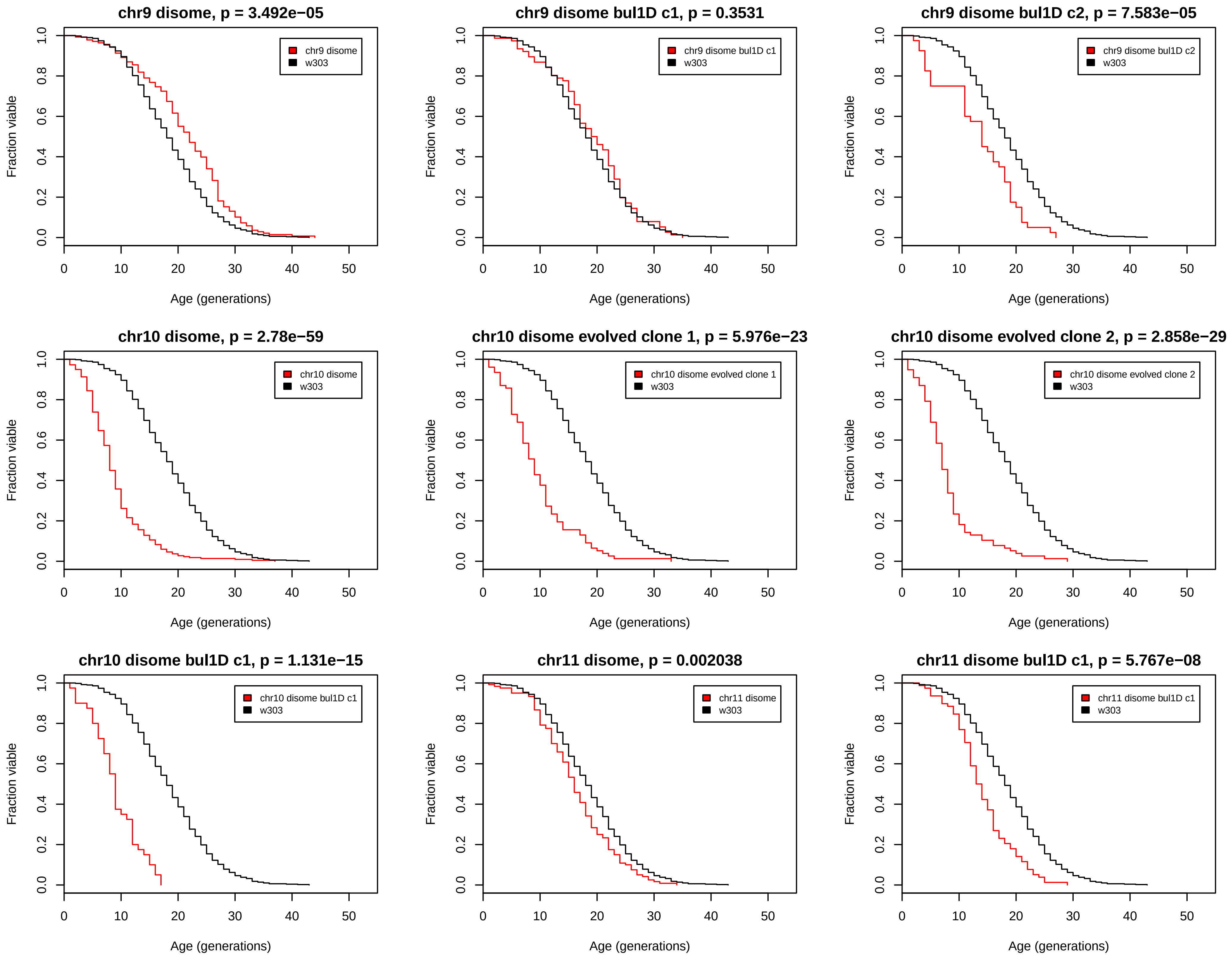
chr9 disome, p = 3.492e−05
chr9 disome
w303
chr9 disome bul1D c1, p = 0.3531
chr9 disome bul1D c1
w303
chr9 disome bul1D c2, p = 7.583e−05
chr9 disome bul1D c2
w303
chr10 disome, p = 2.78e−59
chr10 disome
w303
chr10 disome evolved clone 1, p = 5.976e−23
chr10 disome evolved clone 1
w303
chr10 disome evolved clone 2, p = 2.858e−29
chr10 disome evolved clone 2
w303
chr10 disome bul1D c1, p = 1.131e−15
chr10 disome bul1D c1
w303
chr11 disome, p = 0.002038
chr11 disome
w303
chr11 disome bul1D c1, p = 5.767e−08
chr11 disome bul1D c1
w303
Fraction viable
0.0
0.2
0.4
0.6
0.8
1.0
Age (generations)
0
10
20
30
40
50

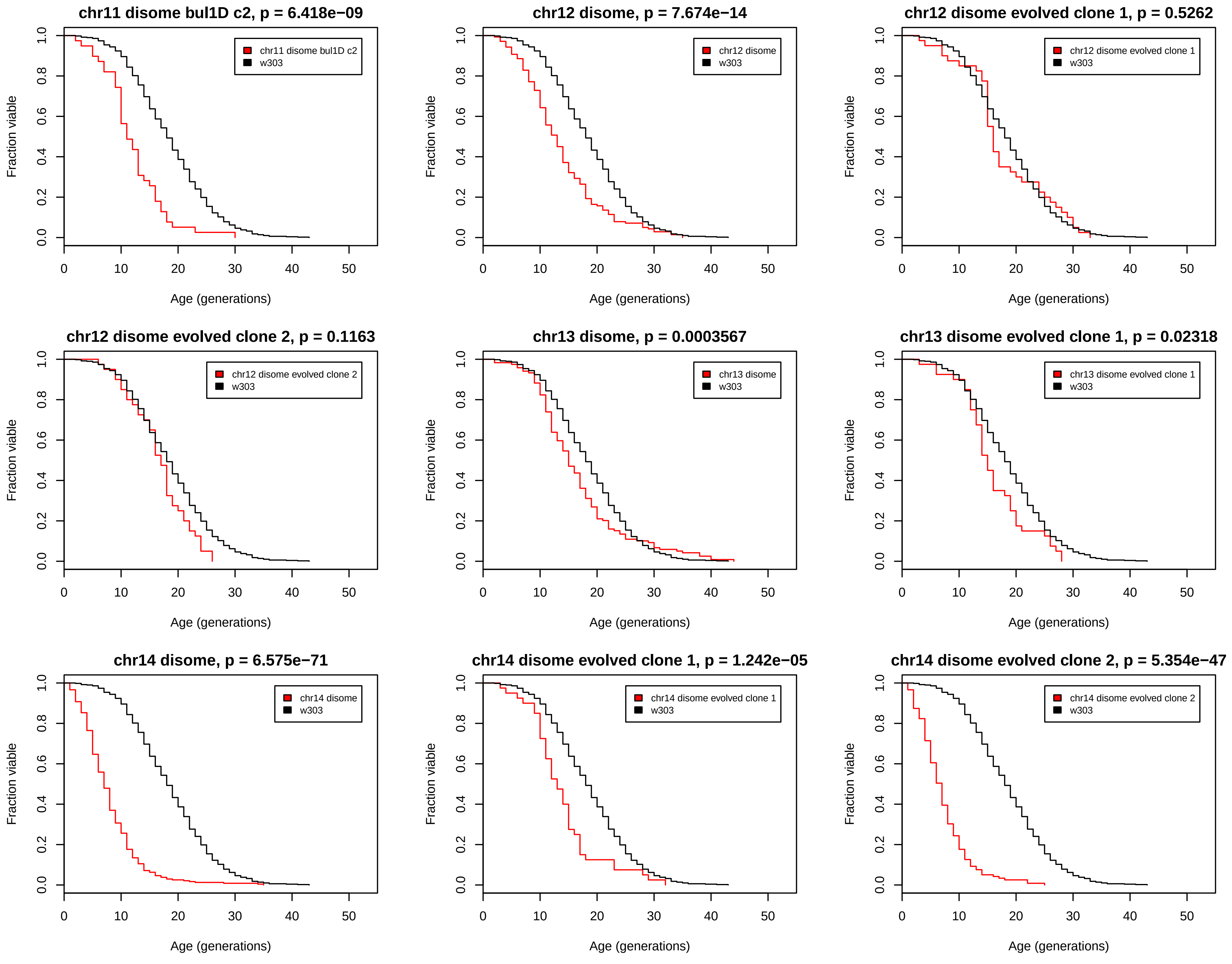

chr11 disome bul1D c2, p = 6.418e−09
chr11 disome bul1D c2
w303
chr12 disome, p = 7.674e−14
chr12 disome
w303
chr12 disome evolved clone 1, p = 0.5262
chr12 disome evolved clone 1
w303
chr12 disome evolved clone 2, p = 0.1163
chr12 disome evolved clone 2
w303
chr13 disome, p = 0.0003567
chr13 disome
w303
chr13 disome evolved clone 1, p = 0.02318
chr13 disome evolved clone 1
w303
chr14 disome, p = 6.575e−71
chr14 disome
w303
chr14 disome evolved clone 1, p = 1.242e−05
chr14 disome evolved clone 1
w303
chr14 disome evolved clone 2, p = 5.354e−47
chr14 disome evolved clone 2
w303
Fraction viable
Age (generations)
0.0
0.2
0.4
0.6
0.8
1.0
0
10
20
30
40
50

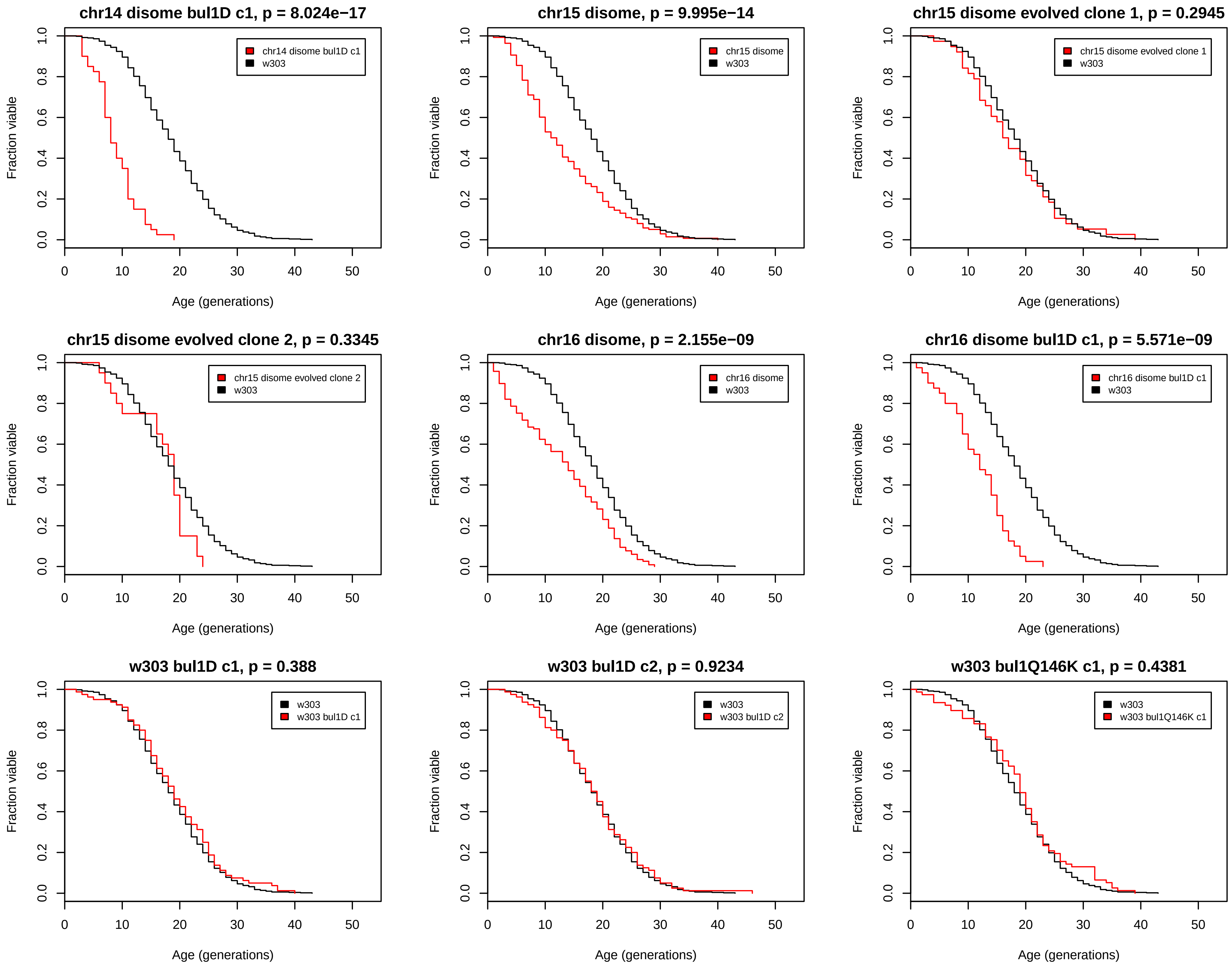
chr14 disome bul1D c1, p = 8.024e−17
chr14 disome bul1D c1
w303
chr15 disome, p = 9.995e−14
chr15 disome
w303
chr15 disome evolved clone 1, p = 0.2945
chr15 disome evolved clone 1
w303
chr15 disome evolved clone 2, p = 0.3345
chr15 disome evolved clone 2
w303
chr16 disome, p = 2.155e−09
chr16 disome
w303
chr16 disome bul1D c1, p = 5.571e−09
chr16 disome bul1D c1
w303
w303 bul1D c1, p = 0.388
w303
w303 bul1D c1
w303 bul1D c2, p = 0.9234
w303
w303 bul1D c2
w303 bul1Q146K c1, p = 0.4381
w303
w303 bul1Q146K c1
Fraction viable
0.0
0.2
0.4
0.6
0.8
1.0
Age (generations)
0
10
20
30
40
50

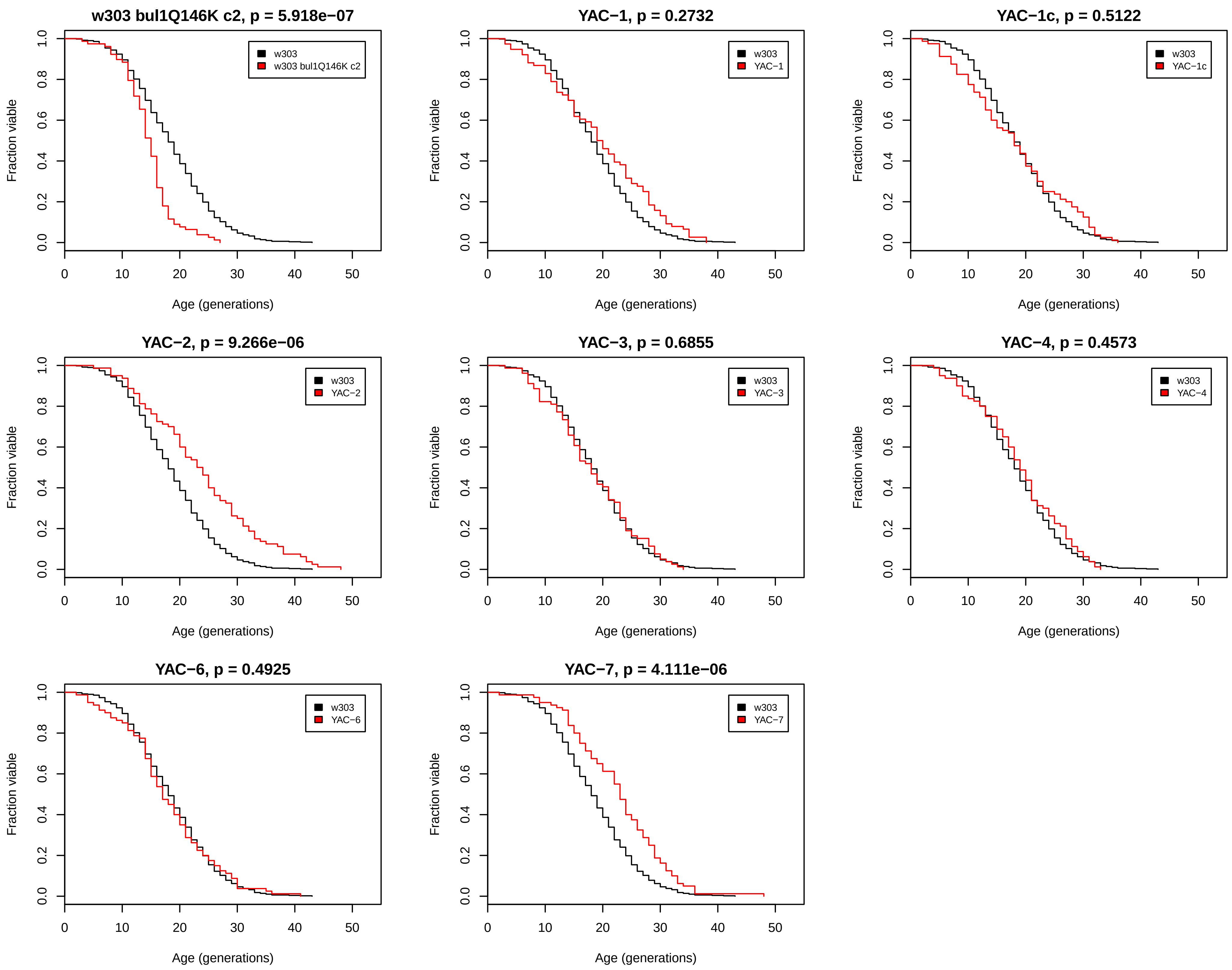
w303 bul1Q146K c2, p = 5.918e−07
w303
w303 bul1Q146K c2
YAC−1, p = 0.2732
w303
YAC−1
YAC−1c, p = 0.5122
w303
YAC−1c
YAC−2, p = 9.266e−06
w303
YAC−2
YAC−3, p = 0.6855
w303
YAC−3
YAC−4, p = 0.4573
w303
YAC−4
YAC−6, p = 0.4925
w303
YAC−6
YAC−7, p = 4.111e−06
w303
YAC−7
Fraction viable
0.0
0.2
0.4
0.6
0.8
1.0
Age (generations)
0
10
20
30
40
50
