# Supplementary figures and images for: Aneuploidy shortens replicative lifespan in Saccharomyces cerevisiae
Source: Aging Cell. 2016 Jan 13;15(2):317–24. doi: 10.1111/acel.12443 (PMC4783355; doi:10.1111/acel.12443)

A

Median Chr12 size = ~2000 kb ( $R^2 = 0.19$ )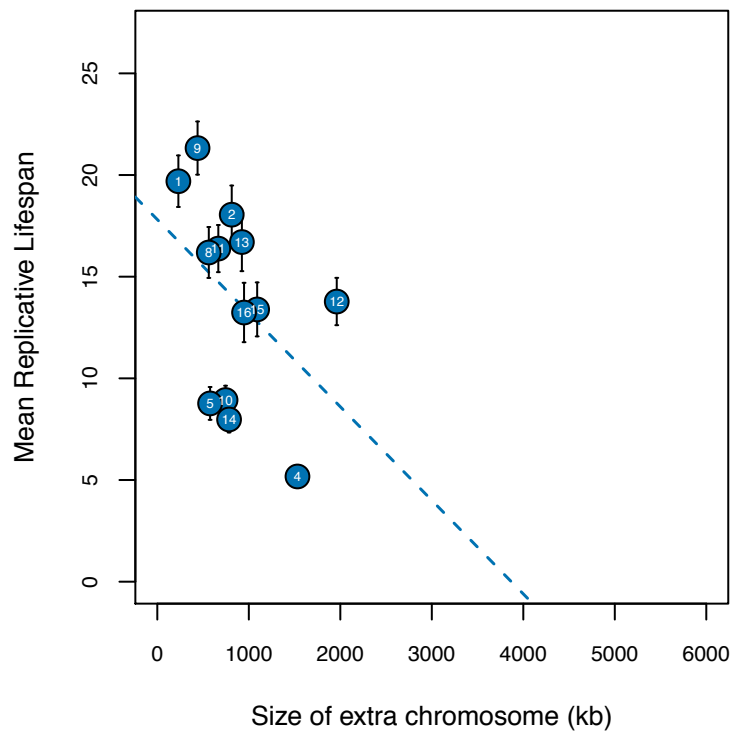

B

Minimum Chr12 size = ~1600 kb ( $R^2 = 0.26$ )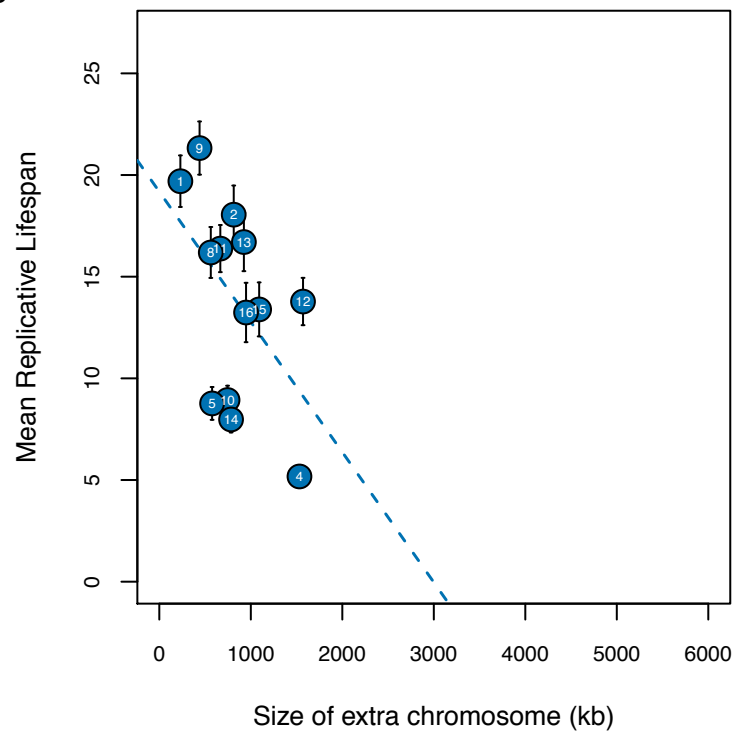

C

Maximum Chr12 size = ~6000 kb ( $R^2 = 0.02$ )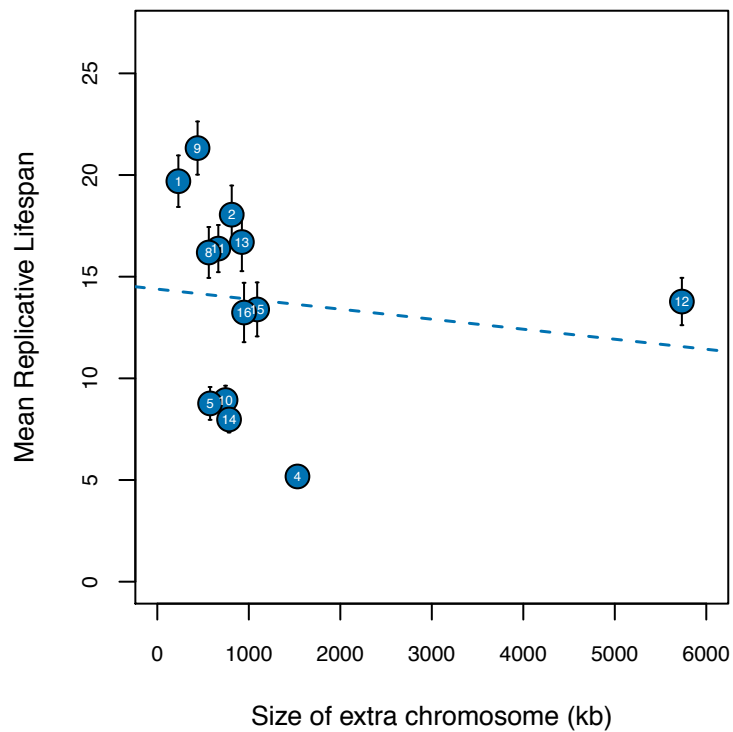

D

Correlation without Chr12 ( $R^2 = 0.38$ )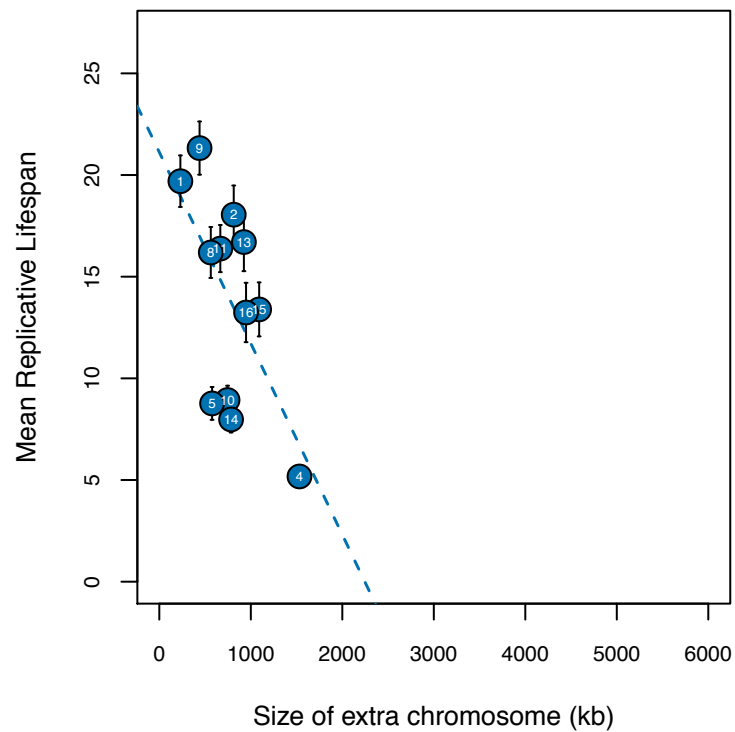

Supplement: Supplementary file 2 — Fig. S2 The estimated size of chromosome 12 affects the correlation between disome size and mean RLS. The median, minimum, and maximum size of chromosome 12 as observed by (James et al., 2009) are plotted in S3A, S3B, and S3C respectively. A duplicate of the disome data presented in Fig. 1C is reproduced in S3D for comparison: in this case chr12 disome strain was removed from the comparison. [file ACEL-15-317-s002.pdf]

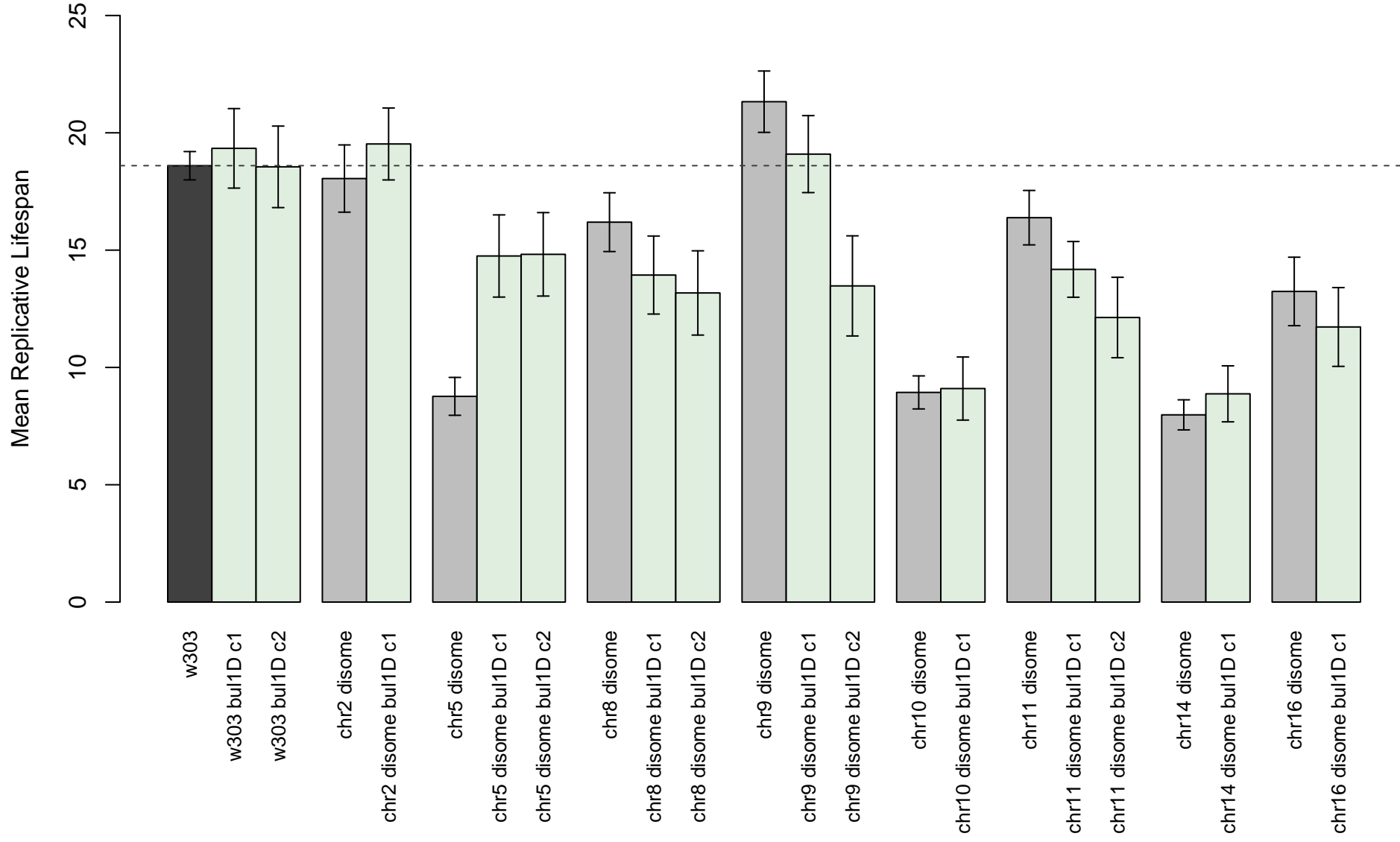

Supplement: Supplementary file 3 — Fig. S3 The lifespan extension seen in chr5 disomes with bul1Δ is not generalizable to other disomic backgrounds. Each bar represents the mean replicative lifespan ± 95% CI. All lifespans were performed on MATa clones except for chr 10 disome bul1Δ which is MATα; there is typically no difference in lifespan between MATa and MATα clones (Kaeberlein et al., 2005), Dark grey = WT control, light grey = disome, mint green = disome, bul1Δ. [file ACEL-15-317-s003.pdf]
